# How does your crystal grow? A commentary on Burton, Cabrera and Frank (1951) ‘The growth of crystals and the equilibrium structure of their surfaces’

**DOI:** 10.1098/rsta.2014.0230

**Published:** 2015-04-13

**Authors:** D. P. Woodruff

**Affiliations:** Department of Physics, University of Warwick, Coventry CV4 7AL, UK

**Keywords:** crystal growth, screw dislocations, two-dimensional nucleation, surface roughening, equilibrium crystal shape, surface steps

## Abstract

The key ideas presented in the classic paper ‘The growth of crystals and the equilibrium structure of their surfaces’ by W. K. Burton, N. Cabrera and F. C. Frank, published in *Philosophical Transactions A* in 1951, are summarized and put in the context of both the state of knowledge at the time of publication and the considerable amount of work since that time that has built on and developed these ideas. Many of these developments exploit the huge increase in the capabilities of computer modelling that complement the original analytic approach of the paper. The dearth of relevant experimental data at the time of the original publication has been transformed by the application of increasingly sophisticated modern methods of surface science. This commentary was written to celebrate the 350th anniversary of the journal *Philosophical Transactions of the Royal Society*.

## 1. The publication and contemporary work

The paper published by Burton, Cabrera and Frank in *Philosophical Transactions A* in 1951 (hereafter referred to as BCF) [1] represented a revolutionary advance in the theory of crystal growth, applying simple methods of statistical mechanics in the treatment of surface fluctuations by Keith Burton and Nicolás Cabrera, and including one key conceptual leap in this field, namely Charles Frank's recognition of the effect of crystal dislocations on crystal growth. Simple metrics provide some measure of its impact: at the time of writing this review, Google Scholar attributes it with 4600 citations, including some 130 in 2013, 62 years after its publication.

A crystalline solid contains molecules (or atoms) arranged on a regular periodic three-dimensional lattice, and can be grown from its vapour or solution by the addition of further molecules arriving at the surface and adopting empty lattice sites. The process can occur if the free energy of molecules in the solid is lower than that in the gas phase or in solution. The basic ideas underlying this process were proposed by Gibbs in 1878 [2] and further developed by Stranski [3] and Becker & Döring [4] in the 1920s and 1930s. Ivan Stranski had played a leading role in the early development of ideas of crystal growth and an interesting personal review of work in the period from 1927 to 1935 has been given by his one-time student and assistant, Rostislaw Kaischew [5]. A key problem recognized in crystal growth was that if the crystal has a perfect surface, terminated by a complete and molecularly smooth layer of atoms, it is generally *not* energetically favourable to attach a single molecule, which has a far smaller number of neighbouring (bonding) molecules than those in the bulk crystal. The key problem for growth to occur is to achieve a two-dimensional nucleus—i.e. a small monolayer-thick island in which the molecules occupy adjacent lattice sites—that is of sufficient size for it to become energetically favourable to add new molecules to the edge of the island and thus to grow a complete new layer on the crystal. The average free energy of the molecules in the island is still higher than that of the molecules in the underlying crystal surface, because molecules at the edge of the island still have fewer near-neighbours, but the energy cost per molecule of creating the island becomes lower and lower as the fraction of edge molecules decreases with increasing island size. Creating this initial nucleus should require a high degree (approx. 50%) of supersaturation in the phase from which growth occurs, supersaturation defining the amount by which molecules in the bulk solid are energetically favoured over the molecules in solution. In practice, this prediction is found to be incorrect: crystals can grow at a significant rate even at low levels (approx. 1% or less) of supersaturation. This is the basic problem addressed in this classic publication of BCF, a four-part paper that brings together and expands ideas developed in the previous 2–3 years by these authors, much of which was presented or discussed at a Faraday Society Discussion meeting [6–8] held at the authors' home institution of the University of Bristol, UK, in April 1949.

**Figure 1.**
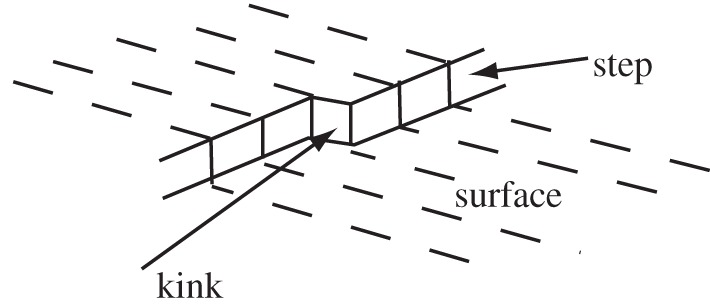
Schematic showing the presence of a kink on a single atomic layer step, effectively treating individual atoms as cubes.

Of course, if one has a single molecular layer step on the surface, this problem is (largely) overcome. Attaching molecules to a step-edge such that the shape of the step is retained involves no extra energy cost. In particular, if the step contains *kinks* (figure 1) (an idea previously introduced by Kossel [9] and Stranski [3]) then adding a molecule at the kink effectively shifts the kink along the step but does not change the number of corner-site molecules and therefore incurs no extra energy cost. Moreover, Frenkel [10] showed in 1945 that such a monolayer step will roughen to contain a significant number of such kinks in thermal equilibrium at quite modest temperatures; indeed, Burton & Cabrera [6] and BCF showed that the equilibrium concentration of these kinks is predicted to be even larger than estimated by Frenkel. In Part I of BCF the rate of advance of such steps and their crystallographic orientation dependence in crystal growth is evaluated.

This idea provides a mechanism for growth at low supersaturation of a crystal that presents a stepped surface, but if the surface between the steps is molecularly smooth (i.e. it has a low Miller index and is thus relatively close-packed), growth will stop when all the steps have propagated to the edge of the crystal, leaving a perfectly smooth surface containing no step or kink sites for further growth. Frenkel suggested that this problem may be overcome by the thermal roughening of the surface to produce a significant equilibrium concentration of steps on a surface, but BCF show clearly that this could only occur at temperatures very close to (or above) the melting point. Note that, at an orientation of high Miller indices, a surface is intrinsically stepped or ‘rough’ on a molecular scale to offer a continued supply of step and kink sites; however, during growth, crystals generally become dominated by the low-index ‘step-less’ orientation surfaces with the lowest free energies.

**Figure 2.**
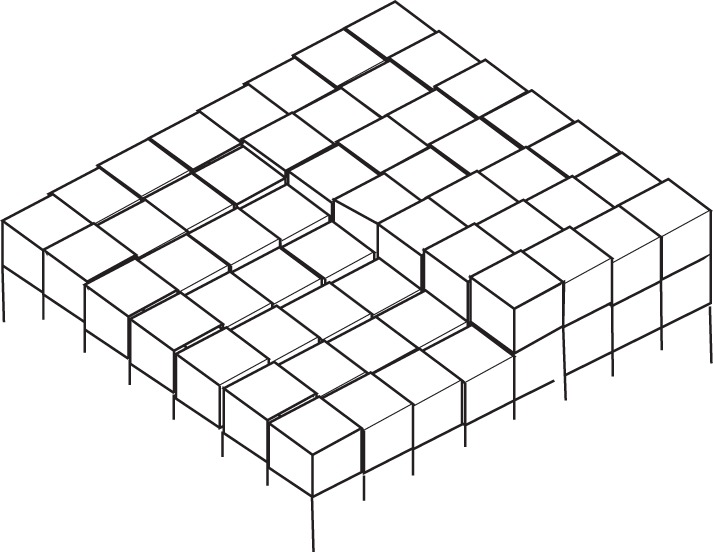
Schematic (‘cubic atom’) diagram showing the intersection of a screw dislocation with a crystal surface.

The key new idea [8,11] developed by BCF is that real solids are generally not perfectly crystalline, but contain dislocations, and in particular the intersection of a screw dislocation with a surface leads to the presence of a single molecular layer step (figure 2) that can never saturate; it allows growth of layers around the point of intersection of the dislocation and the surface without ever growing out of the crystal, like laying a carpet up a spiral staircase.

**Figure 3.**
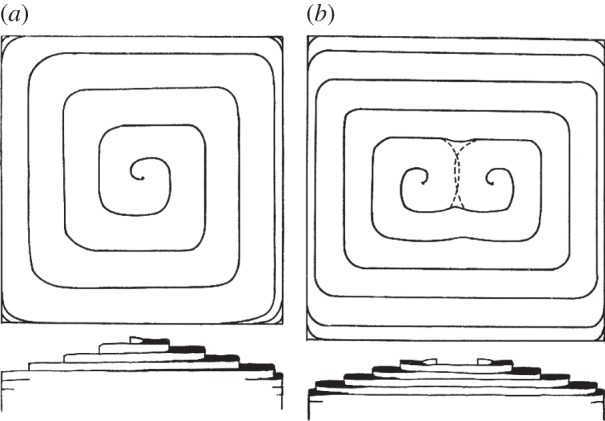
Schematic of growth spirals in plan and side views resulting from growth at the intersection of a single screw dislocation and a crystal surface (*a*) and a pair of such dislocations and a surface (*b*). From BCF [1] (Copyright The Royal Society).

Growth at such an intersection of a single screw dislocation with the crystal surface leads to the presence of growth spirals on the surface, as shown in figure 3, while more complex step patterns at the surface result from combinations of surface/dislocation intersections. Apparently Frank had originally imagined that these spirals were essentially a theoretical concept and was surprised when, at the Faraday Discussion meeting where he first presented the idea [8], a member of the audience announced that he had seen evidence of them experimentally [12]. This first direct evidence of such single molecule-height step patterns on surfaces, obtained in an investigation of natural beryl crystals, was subsequently reported by Griffin [13], who observed them using optical reflection (metallographic) microscopy, the images being enhanced by depositing Ag films on the surface and adjusting the imaging conditions to exploit interference effects at the step edges. Using this approach it was possible to estimate the step heights to be less than 20 Å, although not to show explicitly that they had the height of 7.9 Åexpected for a monomolecular step. However, using the same approach Verma [14] showed that similar growth spirals imaged on a carborundum (SiC) surface had heights measured to be in the range 14.1–15.5 Å, fully consistent with the expected monomolecular height of 15.08 Å. Experiments performed in Bristol at this time by Forty [15,16] also demonstrated *in situ* microscopy of growth spirals advancing during growth of cadmium iodide crystals from solution, although in this case much higher steps, associated with screw dislocation groups, were observed. Many examples of the observation of growth spirals were presented at the Bunsen Conference in Berlin, Germany, in 1952, clearly verifying Frank's predictions, although at the time Stranski was apparently loath to recognize their importance [5].

Part II of BCF not only expands on the topological implications of multiple dislocations on the growth patterns, but also quantifies their impact on growth rates and compares these predictions with the (very limited) available experimental data. The key conclusion, of course, is that significant rates of growth can occur at very low levels of supersaturation only in the presence of crystal dislocations.

Parts III and IV of BCF consider the theory of the equilibrium structure of crystal surfaces and its impact on the rates of crystal growth. In Part III the focus is the equilibrium structure of steps and the size and shape of island nuclei required for growth on a perfect crystal surface without dislocations. As part of this discussion they also introduced a two-dimensional version of Wulff's theorem, which states that the shape of a crystal in equilibrium is such that the distances of the faces from the centre of the crystal are proportional to their free surface energies per unit area. This theorem had been stated but not proved by Wulff in 1901 [17], and had been subjected to various attempts to provide a formal proof, notably by von Laue in 1943 [18]. BCF note in an appendix that there was ‘no satisfactory proof of the three-dimensional Wulff theorem’ even then, but do offer a proof of the two-dimensional case. In fact papers published soon afterwards by Conyers Herring [19,20] are generally attributed with addressing the three-dimensional problem, and certainly broaden the range of applications of the theorem.

The final part (IV) of BCF addresses the suggestion of Frenkel that a significant thermal equilibrium concentration of steps on an otherwise flat surface should occur in the same way as that of kinks on steps, thus ensuring step growth can always occur even on low-index surfaces. Specifically, they address the question of the structure, and particularly the roughness, of a crystal surface *as a cooperative phenomenon*. They remark that ‘it seems to be characteristic of cooperative problems that the thermodynamic functions are non-analytical functions of the temperature; they thus possess discontinuities or infinities in themselves or their derivatives’. Specifically, they found that there is a well-defined transition temperature in the surface roughness parameter. Their results show that for close-packed surfaces in which atoms have many nearest neighbours this roughening transition temperature is close to, or above, the bulk melting temperature. Thus, growth on such surfaces can only occur at reasonable temperatures and low supersaturation with the aid of dislocation-derived steps. This is the key conclusion mentioned above.

Although not directly related to the BCF paper and its ideas, this historical period was also one in which modern-day ideas on epitaxial growth—the growth of thin films of the same or a different material (‘homoepitaxy’ or ‘heteroepitaxy’) on crystalline surfaces that are either lattice-matched or have a well-defined crystallographic relationship with the growing film—were founded. In particular, a pair of papers by Frank and his student at that time, Johannes van der Merwe [21,22], defined one (‘layer-by-layer’) mechanism of epitaxial growth in which each layer is completed before the next is nucleated; this has subsequently become known as Frank–van de Merwe growth in the classification of such mechanisms [23]. The competing mechanisms are named Volmer–Weber growth (in which the overlayer grows as three-dimensional islands on the underlying crystal) and Stranski–Krastanov growth [24] (in which the film grows as three-dimensional islands on top of a first covering layer).

Not only did these papers lay down the foundations of modern understanding and quantifications of crystal growth and the associated kinetics, but the idea of a surface roughening transition has also been a source of significant subsequent interest in its own right.

## 2. Developments in crystal growth theory and experiments

The key qualitative prediction of BCF, the occurrence of growth spirals on growing and grown surfaces due to the role of screw dislocations in the growth, continues to attract interest even now. New examples of observations continue to be reported, though the experimental methods now available are far more precise. In particular, both scanning tunnelling microscopy (STM) and atomic force microscopy (AFM) provide the means to obtain microscopic images of surfaces with atomic resolution and to measure step heights with picometre resolution. Very recent examples include AFM observations of single-layer step spirals on GaN(0001) [25] and a review of similar observations in both growth and dissolution of some mineral surfaces [26]. However, detailed analysis of AFM images of single-layer spirals and loops on l-cystine crystal surfaces has been interpreted as inconsistent with BCF [27]; the authors argue that in these crystals of complex chiral molecules the standard BCF theory, based on so-called Kossel crystals (simple cubic–nearest-neighbour bonding), is no longer directly applicable. The development of these atomic resolution scanning probe methods has also led to direct observations of kinks on steps and experimental estimates of their formation energy, as in a study of gypsum surfaces [28].

More generally, it took a decade or more for other aspects of BCF to impact more fully on the crystal growth community. Crystal growers were inclined to view the crystallization process as something of a ‘black box’ and there was a large cultural divide between them and the theoretical physicists who were familiar with the methods of statistical mechanics on which much of the original BCF publication was based [12]. Increasingly, however, BCF and advances in crystal growth theory have developed in conjunction with experimental data on growth kinetics, very little of which was available at the time of the publication of BCF. Two particular aspects have had a significant impact on these developments. The first is that it became clear that, despite the widespread evidence for the important role of dislocations in influencing growth, as manifested by the many observations of growth spirals in surfaces and interfaces, this is not the *only* mechanism of growth. In particular, driven by the need to produce highly perfect semiconductor crystals for applications in electronics (defects acting as scattering centres that degrade their electrical properties), it was found to be possible to grow large crystals of silicon, in particular, that are dislocation-free. Most commonly such growth has been from the melt (e.g. [29–31]), but it has also been shown to be possible using molecular beam epitaxy (MBE). MBE is an ultra-high-vacuum (UHV) technique in which controlled growth of ultrathin films of high perfection can be achieved, particularly valuable for the fabrication of modern semiconductor devices (e.g. [32–34]) including not only two-dimensional films but also one-dimensional wires and (nominally) zero-dimensional ‘dots’. The fact that such crystals may be free of dislocations clearly implies that spiral growth at screw dislocations does not play a role, and in general the growth is based on the generation of single-layer height two-dimensional nuclei followed by lateral expansion of these layers by step-edge growth until each layer is completed. This layer-by-layer or Frank–van der Merwe growth can be clearly identified by monitoring diffracted beam intensities from the surface. Most commonly this is achieved using the technique of reflection high-energy electron diffraction (RHEED), a technique using grazing incidence and grazing emergence of the electrons, thus allowing molecular beam sources to be placed directly in front of the crystal. It is found that the specular diffracted beam intensity oscillates during growth with a period that corresponds to the completion of new atomic or molecular layers on the surface. These modulations are attributed to the alternately atomically smooth and rough surface that occurs during this layer-by-layer growth [35]. These RHEED oscillations provide an *in situ* method of monitoring the progress of the growth of ultrathin films with exquisite precision. Note, though, that as growth proceeds the oscillations damp in amplitude, attributed to an increased roughening due to nucleation of new layers on the islands of incomplete layers; the number of oscillations clearly visible during growth varies from fewer than 10 to many tens depending on the system being studied and the conditions of growth, but a smooth surface can generally be restored by annealing, allowing strong oscillations to be recovered in subsequent growth. Note that in the growth of III–V compounds, such as GaAs, the MBE technique exploits the fact that the sticking factor of the different elemental beams (As_2_ or As_4_ and Ga) depends on the composition of the surface, such that the correct stoichiometry is achieved without having to set the fluxes of the two beams to exactly the same arrival rate. In the case of Si MBE, it is possible to use true molecular beams, most notably exploiting the pyrolysis of silane (SiH_4_) or disilane (Si_2_H_6_) at the surface, although it is now more common to use vacuum evaporation of solid silicon such that the ‘molecular’ beam is actually atomic Si.

The second important development has been the increasing use of computational modelling of crystal growth using Monte Carlo methods, a possibility that did not exist at the time of BCF due to the lack of suitable computational resources. Such calculations are very intensive users of computer time, but the huge increases in available computational power that have occurred in the last few decades make it possible to perform such calculations on larger and larger model systems (increasingly large numbers of atoms or molecules), and to vary the model parameters to understand the dependence of the kinetics and resulting crystal shapes as a function of temperature, supersaturation and binding energy. Two reviews of some of the early development of this work up to the 1980s provide further details [12,36]. In many cases, the underlying models are closely similar to those used to describe the growth kinetics in BCF, such as the assumption that only nearest-neighbour bonding needs to be considered (although BCF included next-nearest-neighbour bonding to treat surface roughening). In the absence of dislocations, these calculations typically show that at low temperatures (small values of *kT*/*ϕ*, where *ϕ* is the energy associated with a single nearest-neighbour bond) growth rates are found to be linear in the chemical potential excess Δ*μ* (effectively the supersaturation) on less well-packed surface orientations, but show very low growth rates on the most close-packed surface orientations at low values of Δ*μ*, with significant growth only occurring at high values of Δ*μ*. At high temperatures, on the other hand, with *kT*/*ϕ*≈1, linear growth kinetics are seen on all faces. This is qualitatively consistent with the predictions of BCF, although it seems that the barrier to island nucleation at the highest temperatures may be somewhat less than that indicated by BCF's simple analytic treatment, on some real materials.

More recently, a number of significant enhancements have been made in the underlying theories on which calculations are based. For example, going beyond simple Kossel crystals leads to the inequivalence of different kink structures on steps that can impact on the growth kinetics [37]. Moreover, BCF effectively assume that growth always takes place on a surface in which the step structure is in equilibrium. This may be adequate at low levels of supersaturation and low growth rates, but this is commonly not the case in MBE growth, a method that has become particularly important in the last few decades as it allows highly controlled production of complex semiconductor device structures. Appropriate theories must therefore take account of the movement of the steps during the arrival of new material from the gas phase and the associated diffusion over the surface (e.g. [38–40]).

## 3. Developments in surface roughening

**Figure 4.**
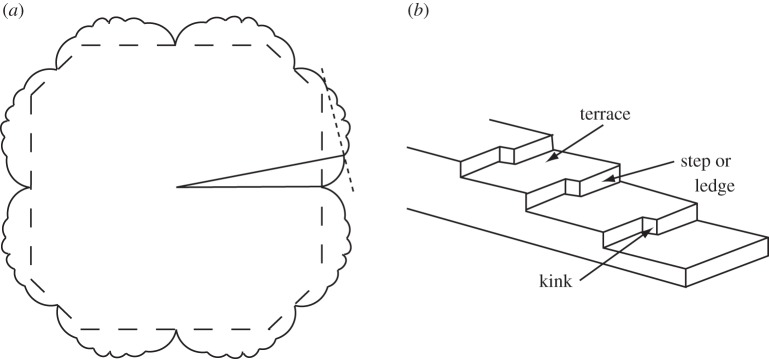
(*a*) The solid line shows an example of a polar plot of the surface free energy per unit area, *γ*, of a crystal as a function of orientation. The dashed lines show the resultant equilibrium shape obtained from the Wulff construction. (*b*) Schematic diagram of the terrace–ledge–kink model of a surface of some arbitrary (high-index) or vicinal orientation.

The phenomenon of surface roughening and its implications raised by BCF have also continued to attract considerable interest, both through new theoretical and computational studies and through experimental investigations. The core finding of BCF was that the roughening transition (above which continuous crystal growth could occur without the need for screw dislocations or island nucleation) may well not occur on low-index close-packed surfaces at temperatures below the melting point, but for other surface orientations that are intrinsically stepped, the situation is different. To understand much of the work in this field since BCF, it is helpful to consider the structure of a surface of arbitrary orientation and the equilibrium shape of a three-dimensional crystal. As remarked above, this equilibrium shape is defined by the Wulff theorem, and figure 4 shows an example of the application of the construction based on this theorem. The figure shows schematically a polar diagram of the surface free energy per unit area of a crystal as a function of orientation. This so-called γ-plot contains cusps associated with local singular minima in the surface free energy at many specific orientations and has been referred to by Frank as a ‘raspberry figure’. The equilibrium shape is the inner envelope of the set of planes drawn perpendicular to radial vectors at the point of intersection of the γ-plot. At low temperatures, this is typically dominated by facets at low-index orientations corresponding to the deepest cusps in the γ-plot. At higher temperatures, however, the cusps are lost at orientations that are above their roughening temperature, while the cusps at the main facet orientations become less deep, and smoothly rounded corners may appear on the equilibrium shape. Rounded sections of the equilibrium shape thus indicate that these orientations are above their roughening temperature. However, the absence of these orientations on the equilibrium shape does not necessarily imply that they would not be above their roughening temperature; such an absence simply means that they are unstable to faceting to a different orientation or, in the case of an extended surface rather than a small particle, that they are unstable to a transition to a ‘hill and valley’ structure of other orientations that do appear on the equilibrium shape [19]. Note that surface orientations that are not close-packed with low Miller indices, and particularly ‘vicinal’ surfaces that are close to these low-index orientations, are usually envisaged in terms of periodic arrays of steps or ledges and kinks superimposed on low-index terraces; this terrace–ledge–kink model is also shown in figure 4.

Early evidence of a distinct roughening temperature, even on low-index faces, was obtained from a study of the growth of crystals of C_2_Cl_6_ and NH_4_Cl from the vapour at different temperatures, a rather sharp change in the growth morphology from faceted to dendritic (the dendrites having rounded surfaces) being found for C_2_Cl_6_ at a growth temperature of approximately 100°C [41]. At elemental metal surfaces—systems more nearly matched to the underlying models used in BCF—the experimental evidence is consistent with no roughening of the most close-packed surfaces, even at temperatures very close to the melting point. For example, electron microscopy studies of small (approx. 5 μm) particles of Pb (melting point, *T*_m_=327°C) deposited on graphite clearly show (111) and (100) facets on the equilibrium shapes at temperatures up to 300°C [42]. More recent studies, particularly of stepped surfaces, have mostly exploited modern surface science techniques including STM, but also surface diffraction using thermal-energy helium atoms and X-rays. For example, helium atom scattering was performed from stepped ((113), (115) and (117)) surfaces of Cu at various temperatures and the temperature dependence of the diffracted intensities interpreted in terms of roughening transitions occurring at temperatures in the range approximately 0–240°C depending on the step density [43]. X-ray scattering from Cu(113) led to similar conclusions [44], identifying the roughening transition temperature as 620±10 K or approximately 0.46*T*_m_.

Similar experiments performed on (110) and (100) faces of Cu led to a recognition of more complex behaviour. The face-centred-cubic (110) surface is interesting in that it is significantly less close-packed than the (111) and (100) surfaces, so it seems a possible candidate for a roughening transition below the melting point, and the results of initial diffraction studies were interpreted in terms of such a transition. However, later work demonstrated that the sharp change in diffracted beam intensities at higher temperatures could be attributed to enhanced anharmonicity in the surface vibrations rather than a true roughening transition [45]. Further complications in interpreting such experiments, including the occurrence of thermally induced reconstructions to a different ordered phase on some low-index surfaces, are discussed in several reviews [46,47]. In studies of stepped or vicinal surfaces, of course, the atomic resolution real-space imaging capability offered by STM has strongly complemented the surface diffraction studies and led to a large number of investigations of step-related phenomena on surfaces, allowing experimental determinations of step and kink energies and diffusion activation energies that could not have been imagined at the time of publication of BCF (e.g. [48]).

## 4. Surface melting

One further phenomenon in the context of a transition at the surface from order to disorder, not discussed by BCF (and not relevant to crystal growth), which has attracted interest both experimentally and theoretically, is surface melting. It is well known that it is relatively easy to cool a liquid quite far below the bulk melting temperature before solidification occurs; this occurs because of the need to overcome the excess energy of the solid–melt interface of a solid nucleus, which can only be achieved by a substantial degree of undercooling. The problem is the three-dimensional equivalent of the surface island nucleation problem in crystal growth addressed by BCF. By contrast, it is generally *not* possible to superheat a solid above its melting point. The reason seems to be that melting can occur at the surface without having to overcome this energy barrier, and a very simple thermodynamic argument supports the idea that melting of a surface (i.e. of the outermost several atomic layers of a surface) could occur below the bulk melting temperature. In particular, melting at a surface replaces a solid–vacuum interface by a solid–melt interface and a melt–vacuum interface, but the sum of the free energies associated with these two interfaces, *γ*_sm_+*γ*_mv_, is commonly less than that of the single solid–vacuum interface, *γ* (or *γ*_sv_). Melting may, therefore, be energetically favoured at a temperature below the bulk melting point, despite the fact that the free energy of the melt phase is slightly higher than that of the solid ([49] and references therein). Evidently, in this simple picture, the thickness of the melted layer would increase as the temperature gets closer and closer to the bulk melting temperature. Of course, this simple argument ignores any possible interaction between the two interfaces.

Note that surface melting is distinct from surface roughening. In surface roughening, it is the occupation of lattice sites at different heights above the surface that becomes disordered; in surface melting, it is the lateral order (and ultimately also the order perpendicular to the surface) that is lost. Direct experimental evidence for the existence of surface melting was first achieved through the use of medium energy ion scattering (MEIS—using H^+^ ions at energies of approx. 100 keV), initially from Pb(110). MEIS provides a measure of how many atoms are displaced from the ordered lattice of a solid, and the results showed clearly a very pronounced enhancement of this number for temperatures more than 20°C below the bulk melting temperature [50]. Subsequent measurements showed that there was no such effect on Pb(111), but there was on a range of surfaces vicinal to Pb(111). The results were shown to be fully consistent with the simple interfacial energy arguments outlined above, the solid–vacuum interfacial energy at the most closely packed (111) surface being sufficiently low for surface melting not to be energetically favoured at this orientation [51]. Note that, although MEIS is also sensitive to enhanced vibrational amplitudes (and anharmonicity) with increasing temperature, it should not be sensitive to surface roughening.

## 5. Conclusion

The key ideas and conclusions of BCF continue to be relevant to research today, not only to crystal growth but also to many aspects of the structure and dynamics of surfaces. BCF was originally directed particularly to crystal growth from solution, for which there was relatively more experimental data at the time. Nowadays much theoretical, computational and experimental work focuses on epitaxial growth from the vapour phase, using MBE or chemical vapour deposition. The wealth of new data from experiments on, and computational simulations of, these growth techniques, and a more widely understood recognition of the relevance of these two complementary approaches, has brought theory and experiment far closer together than in the 1950s. The massive expansion in available techniques to study the structure and dynamics of well-characterized surfaces that began in the 1970s and continues to this day has also opened up new insights into surface phase transitions and the mechanisms and kinetics of crystal growth. Inevitably BCF did not address many of the newly found complications, yet the simple models and ideas that were developed by them continue to impact on current research in these fields.

## Author profile

**Phil Woodruff** is Emeritus Professor of Physics at the University of Warwick with a continuing interest in the structure and electronic and chemical properties of well-characterized solid surfaces. He first gained new insights from reading BCF's paper during his PhD studies on solidification and melting, but he also returned to the paper more recently when studying atomically stepped surfaces using scanning tunnelling microscopy. Charles Frank not only lectured Phil while he was an undergraduate at the University of Bristol, but also later acted as external examiner of his PhD thesis at Warwick; Phil remembers him as an imposing figure with a sharp mind and an exceptional ability to think in three dimensions.
